# Supplementary material for: Engagement in leisure activities and depression in older adults in the United States: Longitudinal evidence from the Health and Retirement Study
Source: Soc Sci Med. 2022 Feb;294:114703. doi: 10.1016/j.socscimed.2022.114703 (PMC8850653; doi:10.1016/j.socscimed.2022.114703)
Supplement: Multimedia component 1 [file mmc1.docx]

## Supplementary Material

### Sample selection


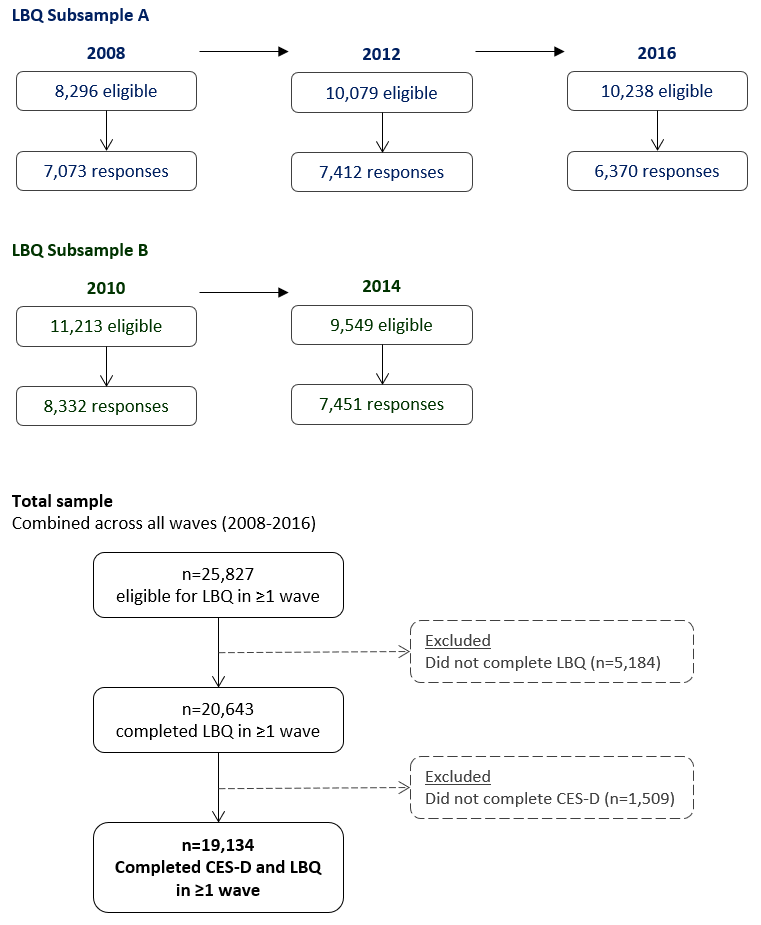


*Figure S1.* Flowchart illustrating the sample selection process. LBQ: Leave-Behind psychosocial and lifestyle Questionnaire (which included questions on leisure activities). CES-D: Center for Epidemiologic Studies Depression scale. At each HRS wave, a rotating random 50% subsample of participants were given the LBQ to complete and return by mail. Participants were eligible to complete the LBQ in either 2008, 2012, and 2016 or in 2010 and 2014. Our final analytical sample included participants who completed both the LBQ and CES-D in at least one wave (n=19,134; 33,880 observations; 1-3 [mean=1.8] repeated measures per person).

### Associations between confounders and engagement in leisure activities

Table S1. *Ordinal logistic regressions testing the associations between potential confounders (exposures) and frequency of engagement in leisure activities (outcomes) at baseline.*

|  | **Reading** | **Writing** | **Baking/cooking** | **Sewing** | **Hobby/project** | **Club** | **Organization** |
| --- | --- | --- | --- | --- | --- | --- | --- |
| **Confounder** | OR (95% CI) | | | | | | |
| Gender | **0.61 (0.54-0.70)** | **0.47 (0.43-0.51**) | **0.25 (0.23-0.27)** | **0.05 (0.04-0.07)** | **1.23 (1.13-1.33)** | **1.10 (1.01-1.21)** | **0.89 (0.80-1.00)** |
| Race/ethnicity: Black | 1.02 (0.84-1.23) | 1.04 (0.92-1.19) | 0.96 (0.84-1.09) | **0.58 (0.47-0.71)** | **0.60 (0.53-0.69)** | 0.88 (0.77-1.02) | **1.56 (1.34-1.83)** |
| Race/ethnicity: Other | 0.76 (0.60-0.97) | 0.91 (0.74-1.11) | 1.19 (0.99-1.43) | **1.51 (1.18-1.94)** | **0.81 (0.67-0.97)** | 0.91 (0.74-1.12) | 0.91 (0.70-1.19) |
| Education: high school | **2.31 (1.98-2.69)** | **1.91 (1.67-2.20)** | 1.02 (0.91-1.15) | 1.06 (0.89-1.26) | **1.54 (1.36-1.74)** | **1.98 (1.72-2.29)** | **1.50 (1.25-1.79)** |
| Education: college | **3.57 (2.85-4.48)** | **3.08 (2.62-3.63)** | 1.02 (0.88-1.18) | 1.06 (0.85-1.32) | **1.95 (1.68-2.26)** | **2.69 (2.28-3.18)** | **2.21 (1.80-2.71)** |
| Education: postgraduate | **6.50 (4.55-9.29)** | **6.02 (4.98-7.28)** | 1.05 (0.88-1.26) | 0.93 (0.71-1.23) | **2.04 (1.72-2.42)** | **3.02 (2.49-3.66)** | **3.11 (2.48-3.89)** |
| Age: 60-69 | **1.37 (1.16-1.62)** | 1.11 (0.99-1.25) | **0.86 (0.77-0.96)** | 1.10 (0.93-1.30) | 1.04 (0.93-1.15) | 1.11 (0.98-1.25) | **1.21 (1.05-1.41)** |
| Age: 70-79 | **2.15 (1.77-2.61)** | **1.30 (1.14-1.48)** | **0.76 (0.68-0.86)** | 1.04 (0.86-1.25) | **0.83 (0.74-0.94)** | **1.29 (1.13-1.47)** | **1.37 (1.17-1.61)** |
| Age: ≥80 | **2.31 (1.82-2.92)** | **1.76 (1.50-2.06)** | **0.52 (0.45-0.61)** | 1.03 (0.82-1.30) | **0.62 (0.53-0.72)** | **1.33 (1.13-1.56)** | **1.38 (1.13-1.68)** |
| Marital status | **1.27 (1.04-1.55)** | **1.14 (1.00-1.32)** | **1.13 (1.00-1.29)** | 1.01 (0.83-1.24) | **1.17 (1.03-1.33)** | **1.51 (1.32-1.73)** | **1.39 (1.17-1.65)** |
| Employment: not working | 0.93 (0.75-1.15) | 1.02 (0.88-1.19) | **1.47 (1.27-1.70)** | 1.08 (0.88-1.33) | 1.00 (0.87-1.16) | 1.03 (0.87-1.21) | 1.02 (0.83-1.25) |
| Employment: retired | 1.11 (0.93-1.32) | 0.91 (0.81-1.02) | **1.23 (1.10-1.36)** | **1.32 (1.12-1.55)** | **1.51 (1.37-1.68)** | **1.27 (1.13-1.41)** | 1.10 (0.96-1.26) |
| Income: $19,000-$39,999 | **1.36 (1.14-1.62)** | **0.86 (0.75-0.97)** | **1.16 (1.03-1.31)** | 0.91 (0.76-1.08) | 1.09 (0.96-1.23) | **1.41 (1.23-1.62)** | 1.17 (0.99-1.38) |
| Income: $40,000-$79,999 | **1.44 (1.18-1.75)** | **0.86 (0.75-0.98)** | 1.11 (0.97-1.26) | 0.95 (0.79-1.15) | **1.17 (1.03-1.33)** | **1.48 (1.27-1.71)** | **1.22 (1.02-1.46)** |
| Income: ≥$80,000 | **1.80 (1.42-2.28)** | 1.04 (0.89-1.22) | 1.11 (0.96-1.29) | **0.71 (0.57-0.89)** | **1.26 (1.09-1.46)** | **2.04 (1.73-2.40)** | **1.69 (1.39-2.05)** |
| Neighborhood safety | 1.17 (0.96-1.43) | **0.83 (0.71-0.98)** | 0.94 (0.81-1.09) | 0.94 (0.76-1.16) | 1.13 (0.97-1.32) | **1.38 (1.15-1.64)** | 1.12 (0.92-1.37) |
| Social network | **1.33 (1.10-1.62)** | 1.06 (0.93-1.21) | **1.17 (1.04-1.32)** | **1.23 (1.01-1.49)** | **1.22 (1.08-1.37)** | **1.28 (1.12-1.45)** | **1.21 (1.03-1.42**) |
| Difficulty with ADLs | 0.80 (0.67-0.95) | 0.94 (0.82-1.08) | **0.71 (0.63-0.81)** | **0.74 (0.61-0.89)** | **0.73 (0.65-0.83)** | **0.67 (0.58-0.78)** | 0.87 (0.73-1.04) |
| Difficulty with IADLs | 0.52 (0.43-0.62) | **0.81 (0.70-0.94)** | **0.43 (0.38-0.49)** | **0.75 (0.60-0.92)** | **0.71 (0.61-0.81)** | **0.68 (0.58-0.80)** | **0.72 (0.59-0.87)** |
| Chronic health conditions | 0.87 (0.71-1.08) | **0.82 (0.72-0.94)** | 0.97 (0.85-1.10) | 0.94 (0.77-1.14) | **0.81 (0.71-0.91)** | 0.89 (0.78-1.02) | 1.08 (0.92-1.28) |
| Cognition: q2 | **1.37 (1.17-1.60)** | **1.18 (1.04-1.33)** | **1.16 (1.04-1.30)** | 1.20 (1.01-1.43) | **1.13 (1.01-1.26)** | 1.02 (0.91-1.16) | 0.99 (0.85-1.15) |
| Cognition: q3 | **1.67 (1.37-2.04)** | **1.38 (1.20-1.58)** | **1.18 (1.04-1.34)** | 1.24 (1.02-1.51) | **1.24 (1.10-1.41)** | 1.10 (0.96-1.27) | 1.00 (0.84-1.20) |
| Cognition: q4 | **2.07 (1.64-2.61)** | **1.68 (1.46-1.94)** | **1.27 (1.11-1.45)** | 1.34 (1.09-1.64) | **1.47 (1.29-1.68)** | **1.20 (1.04-1.39)** | 1.15 (0.96-1.39) |

*Note.* N=14,255. As only 50% of the HRS sample completed the LBQ at each wave, baseline statistics were combined across 2008 and 2010, with wave dependent on the subsample that participants were in. Results weighted and based on 20 multiply imputed data sets. A cut-off of three or more on the CES-D was used to indicate depression. Reference categories were as follows: gender female; race/ethnicity White; education none; age 50-59; marital status married; employment status employed; household income <$19,000; neighborhood safety excellent/good; social network less diverse; difficulty with ADLs/IADLs none; chronic health conditions; none; cognition quartile 1. The Other race/ethnicity category included people who identified as American Indian, Alaskan Native, Asian or Pacific Islander, Hispanic, and Other ethnicities. Bold text indicates p<0.05.

### Complete case analyses

Table S2. *Demographic characteristics of the sample at baseline and percentage of the sample with depression at baseline according to demographic characteristics, for complete cases only.*

|  | **Overall** | **Depression** | |
| --- | --- | --- | --- |
|  |  | **No** | **Yes** |
| **Gender** | | | |
| Women | 54% | 78% | 22% |
| Men | 46% | 84% | 16% |
| **Race/ethnicity** | | | |
| White | 86% | 82% | 18% |
| Black | 9% | 71% | 29% |
| Other | 5% | 69% | 31% |
| **Education** | | | |
| None | 13% | 65% | 35% |
| High School | 54% | 79% | 21% |
| College | 22% | 87% | 13% |
| Postgraduate | 11% | 90% | 10% |
| **Age** | | | |
| 50-59 | 36% | 78% | 22% |
| 60-69 | 34% | 84% | 16% |
| 70-79 | 19% | 81% | 19% |
| ≥80 | 11% | 77% | 23% |
| **Marital status** | | | |
| Married | 64% | 85% | 15% |
| Unmarried | 36% | 72% | 28% |
| **Employment status** | | | |
| Employed | 42% | 87% | 13% |
| Not working | 13% | 61% | 39% |
| Retired | 45% | 83% | 17% |
| **Household income** | | | |
| <$19,000 | 18% | 63% | 37% |
| $19,000-$39,999 | 23% | 77% | 23% |
| $40,000-$79,999 | 28% | 84% | 16% |
| ≥$80,000 | 31% | 90% | 10% |
| **Neighborhood safety** | | | |
| Excellent/good | 92% | 82% | 18% |
| Fair/poor | 8% | 61% | 39% |
| **Social network** | | | |
| Less diverse | 46% | 73% | 27% |
| More diverse | 54% | 86% | 14% |
| **Difficulty with ADLs** | | | |
| None | 86% | 85% | 15% |
| One or more | 14% | 50% | 50% |
| **Difficulty with IADLs** | | | |
| None | 88% | 85% | 15% |
| One or more | 12% | 46% | 54% |
| **Long-term conditions** | | | |
| None | 14% | 88% | 12% |
| One or more | 86% | 79% | 21% |
| **Cognition (quartiles)** | | | |
| 0 | 19% | 70% | 30% |
| 1 | 32% | 79% | 21% |
| 2 | 25% | 84% | 16% |
| 3 | 24% | 86% | 14% |

*Note.* N=11,747. Results weighted. The Other race/ethnicity category included people who identified as American Indian, Alaskan Native, Asian or Pacific Islander, Hispanic, and Other ethnicities.

Table S3. *Frequency of leisure activities at baseline, for complete cases only (n=11,747).*

| **Activity frequency** | **Reading** | **Writing** | **Baking/cooking** | **Sewing** | **Hobby/project** | **Club** | **Organization** |
| --- | --- | --- | --- | --- | --- | --- | --- |
| None | 5% | 61% | 30% | 86% | 42% | 63% | 81% |
| Monthly | 9% | 20% | 29% | 7% | 26% | 22% | 15% |
| Weekly | 86% | 19% | 41% | 7% | 32% | 15% | 4% |

*Note.* Results weighted. Leisure activities were defined as: reading books, magazines, or newspapers; writing (such as letters, stories, or journal entries); baking or cooking something special; making clothes, knitting, or embroidery (sewing); working on a hobby or project; going to a sport, social, or other club; and attending meetings of non-religious organizations (political, community, or other interest groups).

Table S4. *Models testing associations between frequency of leisure activities and the odds of depression, for complete cases only.*

|  | **Concurrent** (n=16,666) | | | | | | **Longitudinal** (n=14,071) | | | | | |
| --- | --- | --- | --- | --- | --- | --- | --- | --- | --- | --- | --- | --- |
|  | **Model 1: Unadjusted** | | | **Model 2: Adjusted** | | | **Model 1: Unadjusted** | | | **Model 2: Adjusted** | | |
|  | OR | 95% CI | p value | OR | 95% CI | p value | OR | 95% CI | p value | OR | 95% CI | p value |
| **Reading** | | | | | | |  |  |  |  |  |  |
| Monthly | **0.68** | **0.58-0.81** | **<0.001** | 0.97 | 0.80-1.17 | 0.721 | **0.72** | **0.59-0.88** | **0.001** | 1.06 | 0.83-1.36 | 0.626 |
| Weekly | **0.55** | **0.48-0.64** | **<0.001** | 0.94 | 0.81-1.10 | 0.460 | **0.53** | **0.45-0.63** | **<0.001** | 0.90 | 0.73-1.12 | 0.354 |
| **Writing** | | | | | | | | | | | | |
| Monthly | 1.06 | 0.96-1.17 | 0.279 | 1.01 | 0.90-1.13 | 0.891 | 0.97 | 0.86-1.09 | 0.590 | 0.89 | 0.78-1.02 | 0.089 |
| Weekly | 0.98 | 0.88-1.1 | 0.746 | 0.99 | 0.87-1.12 | 0.882 | 1.02 | 0.9-1.15 | 0.754 | 1.00 | 0.86-1.16 | 0.990 |
| **Baking/cooking** | | | | | | | | | | | | |
| Monthly | 0.95 | 0.87-1.05 | 0.337 | 0.98 | 0.87-1.09 | 0.682 | 0.95 | 0.85-1.07 | 0.394 | **0.87** | **0.76-1.00** | **0.049** |
| Weekly | **0.87** | **0.78-0.96** | **0.004** | 0.90 | 0.80-1.01 | 0.066 | **0.87** | **0.78-0.97** | **0.016** | **0.83** | **0.73-0.94** | **0.005** |
| **Sewing** | | | | | | | | | | | | |
| Monthly | 1.04 | 0.88-1.22 | 0.637 | 0.89 | 0.75-1.06 | 0.178 | **1.25** | **1.05-1.48** | **0.011** | 1.05 | 0.86-1.29 | 0.602 |
| Weekly | **1.30** | **1.1-1.54** | **0.002** | 1.04 | 0.87-1.25 | 0.636 | **1.27** | **1.06-1.52** | **0.010** | 0.91 | 0.74-1.12 | 0.373 |
| **Hobby/project** | | | | | | | | | | | | |
| Monthly | **0.65** | **0.59-0.71** | **<0.001** | **0.83** | **0.75-0.93** | **0.001** | **0.73** | **0.66-0.81** | **<0.001** | 1.02 | 0.89-1.16 | 0.782 |
| Weekly | **0.64** | **0.58-0.71** | **<0.001** | **0.83** | **0.73-0.93** | **0.001** | **0.60** | **0.53-0.67** | **<0.001** | **0.84** | **0.73-0.96** | **0.012** |
| **Club** | | | | | | | | | | | | |
| Monthly | **0.71** | **0.64-0.78** | **<0.001** | **0.89** | **0.80-0.99** | **0.037** | **0.67** | **0.6-0.74** | **<0.001** | **0.79** | **0.69-0.90** | **<0.001** |
| Weekly | **0.64** | **0.56-0.72** | **<0.001** | **0.83** | **0.72-0.96** | **0.010** | **0.60** | **0.52-0.69** | **<0.001** | **0.78** | **0.67-0.92** | **0.003** |
| **Organization** | | | | | | | | | | | | |
| Monthly | **0.83** | **0.74-0.95** | **0.005** | 0.92 | 0.80-1.05 | 0.202 | **0.82** | **0.71-0.94** | **0.004** | **0.84** | **0.72-0.99** | **0.037** |
| Weekly | 1.15 | 0.94-1.41 | 0.186 | 1.12 | 0.89-1.42 | 0.331 | 1.15 | 0.91-1.45 | 0.257 | 1.08 | 0.82-1.42 | 0.581 |

*Note.* For all activities, no engagement was the reference category. In both concurrent and longitudinal analyses, model 2 was adjusted for gender, race/ethnicity, education, age, marital status, employment status, household income, neighborhood safety, social network, difficulty with ADLs and IADLs, long-term conditions, and cognition. Longitudinal model 2 was additionally adjusted for depression in the previous wave. Results weighted. Bold text indicates p<0.05.

### Interactions

Table S5. *Models testing whether the association between frequency of leisure activities and the odds of depression differs according to gender.*

|  | **Concurrent** (n=19,276) | | | | | | **Longitudinal** (n=16,043) | | | | | |
| --- | --- | --- | --- | --- | --- | --- | --- | --- | --- | --- | --- | --- |
|  | **Model 1: Unadjusted** | | | **Model 2: Adjusted** | | | **Model 1: Unadjusted** | | | **Model 2: Adjusted** | | |
|  | OR | 95% CI | p value | OR | 95% CI | p value | OR | 95% CI | p value | OR | 95% CI | p value |
| **Reading** | 1.03 | 0.92-1.15 | 0.642 | 1.04 | 0.92-1.18 | 0.509 | 1.02 | 0.89-1.17 | 0.769 | 1.00 | 0.86-1.18 | 0.965 |
| **Writing** | 0.96 | 0.87-1.07 | 0.460 | 0.95 | 0.84-1.06 | 0.363 | 1.04 | 0.92-1.17 | 0.513 | 1.07 | 0.93-1.22 | 0.354 |
| **Baking** | **1.32** | **1.20-1.45** | **<0.001** | 1.07 | 0.96-1.18 | 0.219 | **1.17** | **1.05-1.29** | **0.004** | 0.93 | 0.83-1.05 | 0.256 |
| **Sewing** | 1.30 | 0.98-1.71 | 0.064 | 1.12 | 0.83-1.50 | 0.469 | 1.25 | 0.87-1.80 | 0.227 | 0.94 | 0.65-1.36 | 0.754 |
| **Hobby** | 0.93 | 0.84-1.03 | 0.150 | 0.96 | 0.87-1.07 | 0.476 | 0.92 | 0.82-1.03 | 0.129 | 0.93 | 0.82-1.05 | 0.243 |
| **Club** | 1.05 | 0.94-1.17 | 0.424 | 1.05 | 0.93-1.18 | 0.447 | 0.99 | 0.87-1.12 | 0.841 | 0.94 | 0.82-1.08 | 0.368 |
| **Org.** | 0.98 | 0.84-1.15 | 0.820 | 1.00 | 0.85-1.19 | 0.958 | 1.05 | 0.88-1.26 | 0.577 | 1.05 | 0.86-1.29 | 0.621 |

*Note.* Interaction terms are reported (gender*activity), with each activity treated as continuous to indicate whether there was overall evidence for an interaction. Results weighted and based on 20 multiply imputed data sets. Bold text indicates p<0.05.

Table S6. *Models testing whether the association between frequency of leisure activities and the odds of depression differs according to age group.*

|  | **Concurrent** (n=19,276) | | | | | | **Longitudinal** (n=16,043) | | | | | |
| --- | --- | --- | --- | --- | --- | --- | --- | --- | --- | --- | --- | --- |
|  | **Model 1: Unadjusted** | | | **Model 2: Adjusted** | | | **Model 1: Unadjusted** | | | **Model 2: Adjusted** | | |
|  | OR | 95% CI | p value | OR | 95% CI | p value | OR | 95% CI | p value | OR | 95% CI | p value |
| Reading | 1.00 | 0.93-1.06 | 0.908 | 1.00 | 0.93-1.08 | 0.952 | 1.04 | 0.96-1.13 | 0.300 | 1.05 | 0.95-1.15 | 0.352 |
| Writing | 0.97 | 0.92-1.02 | 0.240 | 1.00 | 0.94-1.06 | 0.934 | **0.92** | **0.87-0.99** | **0.018** | 0.94 | 0.88-1.02 | 0.129 |
| Baking | 0.99 | 0.94-1.04 | 0.717 | 1.06 | 1.00-1.12 | 0.051 | 1.05 | 0.99-1.12 | 0.083 | **1.08** | **1.01-1.16** | **0.025** |
| Sewing | 0.98 | 0.90-1.07 | 0.724 | 0.96 | 0.88-1.05 | 0.408 | 0.99 | 0.90-1.08 | 0.757 | 0.99 | 0.89-1.10 | 0.872 |
| Hobby | 0.98 | 0.93-1.04 | 0.602 | 1.00 | 0.94-1.06 | 0.969 | 1.05 | 0.99-1.13 | 0.107 | 1.06 | 0.99-1.14 | 0.106 |
| Club | 1.05 | 0.98-1.12 | 0.141 | 1.02 | 0.96-1.09 | 0.497 | 1.06 | 0.99-1.15 | 0.099 | 1.05 | 0.97-1.14 | 0.238 |
| Org. | 0.94 | 0.86-1.03 | 0.213 | 0.98 | 0.89-1.08 | 0.627 | 1.00 | 0.90-1.11 | 0.993 | 1.04 | 0.92-1.17 | 0.505 |

*Note.* Interaction terms are reported (age group*activity), with both activities and age groups treated as continuous to indicate whether there was overall evidence for an interaction. Results weighted and based on 20 multiply imputed data sets. Bold text indicates p<0.05.

### Alternative CES-D threshold to indicate depression

In our main analyses, we used a cut-off of three or more to indicate the presence of depression on the modified eight-item Center for Epidemiologic Studies Depression Scale (CES-D; Steffick, 2000; Turvey et al., 1999). A cut-off of four or more has also been used to indicate depression (Steffick, 2000), so here we have repeated the main analyses using this threshold.

Table S7. *Models testing associations between frequency of leisure activities and the odds of depression (indicated by a score of 4 or more on the CES-D).*

|  | **Concurrent** (n=19,276) | | | | | | **Longitudinal** (n=16,043) | | | | | |
| --- | --- | --- | --- | --- | --- | --- | --- | --- | --- | --- | --- | --- |
|  | **Model 1: Unadjusted** | | | **Model 2: Adjusted** | | | **Model 1: Unadjusted** | | | **Model 2: Adjusted** | | |
|  | OR | 95% CI | p value | OR | 95% CI | p value | OR | 95% CI | p value | OR | 95% CI | p value |
| **Reading** | | | | | | | | | | | | |
| Monthly | **0.73** | **0.62-0.86** | **<0.001** | 1.00 | 0.83-1.20 | 0.970 | **0.72** | **0.60-0.87** | **0.001** | 1.03 | 0.82-1.30 | 0.782 |
| Weekly | **0.54** | **0.47-0.62** | **<0.001** | 0.93 | 0.80-1.08 | 0.322 | **0.47** | **0.40-0.55** | **<0.001** | **0.80** | **0.66-0.98** | **0.027** |
| **Writing** | | | | | | | | | | | | |
| Monthly | **1.14** | **1.03-1.27** | **0.015** | 1.11 | 0.98-1.25 | 0.092 | 0.98 | 0.87-1.11 | 0.777 | 0.87 | 0.75-1.00 | 0.056 |
| Weekly | 1.10 | 0.98-1.23 | 0.102 | 1.09 | 0.96-1.24 | 0.169 | 0.99 | 0.87-1.13 | 0.880 | 0.93 | 0.80-1.09 | 0.377 |
| **Baking/cooking** | | | | | | | | | | | | |
| Monthly | **0.89** | **0.81-0.99** | **0.031** | 0.93 | 0.83-1.04 | 0.214 | 0.92 | 0.82-1.03 | 0.162 | 0.92 | 0.80-1.05 | 0.209 |
| Weekly | **0.82** | **0.75-0.91** | **<0.001** | **0.89** | **0.79-1.00** | **0.049** | **0.84** | **0.74-0.94** | **0.002** | **0.82** | **0.72-0.94** | **0.005** |
| **Sewing** | | | | | | | | | | | | |
| Monthly | **1.19** | **1.01-1.40** | **0.041** | 1.01 | 0.84-1.21 | 0.911 | 1.20 | 0.99-1.45 | 0.059 | 0.96 | 0.76-1.20 | 0.694 |
| Weekly | **1.31** | **1.10-1.57** | **0.002** | 1.03 | 0.86-1.25 | 0.725 | **1.24** | **1.01-1.51** | **0.038** | 0.86 | 0.68-1.07 | 0.182 |
| **Hobby/project** | | | | | | | | | | | | |
| Monthly | **0.61** | **0.55-0.68** | **<0.001** | **0.79** | **0.70-0.89** | **<0.001** | **0.69** | **0.62-0.78** | **<0.001** | 0.97 | 0.84-1.12 | 0.677 |
| Weekly | **0.62** | **0.55-0.69** | **<0.001** | **0.81** | **0.72-0.92** | **0.001** | **0.60** | **0.53-0.68** | **<0.001** | **0.86** | **0.74-0.99** | **0.040** |
| **Club** | | | | | | | | | | | | |
| Monthly | **0.63** | **0.57-0.70** | **<0.001** | **0.80** | **0.71-0.90** | **<0.001** | **0.66** | **0.58-0.74** | **<0.001** | **0.84** | **0.73-0.97** | **0.019** |
| Weekly | **0.54** | **0.47-0.62** | **<0.001** | **0.70** | **0.60-0.83** | **<0.001** | **0.60** | **0.52-0.70** | **<0.001** | **0.83** | **0.70-0.99** | **0.036** |
| **Organization** | | | | | | | | | | | | |
| Monthly | 0.89 | 0.78-1.02 | 0.091 | 0.97 | 0.83-1.12 | 0.639 | **0.79** | **0.69-0.92** | **0.002** | **0.84** | **0.71-1.00** | **0.049** |
| Weekly | 1.12 | 0.91-1.39 | 0.288 | 1.08 | 0.85-1.38 | 0.506 | 1.17 | 0.92-1.49 | 0.190 | 1.17 | 0.89-1.54 | 0.252 |

*Note.* For all activities, no engagement was the reference category. In both concurrent and longitudinal analyses, model 2 was adjusted for gender, race/ethnicity, education, age, marital status, employment status, household income, neighborhood safety, social network, difficulty with ADLs and IADLs, long-term conditions, and cognition. Longitudinal model 2 was additionally adjusted for depression in the previous wave. Results weighted and based on 20 multiply imputed data sets. Bold text indicates p<0.05.

### Complementary measure of depression

In addition to the CES-D, HRS included another measure of depression in every wave, the Short Form Composite International Diagnostic Interview (CIDI-SF). This scale can be used to determine a probable diagnosis of a major depressive episode according to DSM criteria (Nelson et al., 1998; Steffick, 2000). Respondents are asked screening questions about depressed mood and anhedonia and, if they meet criteria for the intensity and duration of depressed mood or anhedonia experienced, they then respond to seven specific symptom questions. By summing responses to these questions, total possible score ranges from 0 to 7, and a score of three or more is indicative of a diagnosis of depression (Nelson et al., 1998; Steffick, 2000).

Table S8. *Models testing associations between frequency of leisure activities and the odds of depression (indicated by a score of 3 or more on the CIDI).*

|  | **Concurrent** (n=19,274) | | | | | | **Longitudinal** (n=16,042) | | | | | |
| --- | --- | --- | --- | --- | --- | --- | --- | --- | --- | --- | --- | --- |
|  | **Model 1: Unadjusted** | | | **Model 2: Adjusted** | | | **Model 1: Unadjusted** | | | **Model 2: Adjusted** | | |
|  | OR | 95% CI | p value | OR | 95% CI | p value | OR | 95% CI | p value | OR | 95% CI | p value |
| **Reading** | | | | | | | | | | | | |
| Monthly | **0.81** | **0.66-0.99** | **0.039** | 0.94 | 0.75-1.17 | 0.576 | 0.92 | 0.69-1.22 | 0.559 | 1.09 | 0.78-1.51 | 0.614 |
| Weekly | **0.64** | **0.54-0.76** | **<0.001** | 0.89 | 0.73-1.08 | 0.227 | **0.75** | **0.60-0.95** | **0.015** | 1.08 | 0.82-1.41 | 0.582 |
| **Writing** | | | | | | | | | | | | |
| Monthly | **1.14** | **1.01-1.30** | **0.041** | 1.06 | 0.92-1.22 | 0.399 | 1.12 | 0.96-1.31 | 0.149 | 1.01 | 0.85-1.20 | 0.879 |
| Weekly | **1.18** | **1.03-1.35** | **0.015** | 1.08 | 0.93-1.24 | 0.332 | **1.24** | **1.05-1.46** | **0.011** | 1.15 | 0.96-1.37 | 0.131 |
| **Baking/cooking** | | | | | | | | | | | | |
| Monthly | 1.01 | 0.89-1.13 | 0.917 | 0.92 | 0.80-1.05 | 0.192 | 0.97 | 0.83-1.13 | 0.716 | 0.93 | 0.78-1.10 | 0.385 |
| Weekly | **0.87** | **0.77-0.98** | **0.023** | **0.80** | **0.70-0.92** | **0.002** | **0.81** | **0.70-0.95** | **0.007** | **0.80** | **0.67-0.95** | **0.012** |
| **Sewing** | | | | | | | | | | | | |
| Monthly | **1.23** | **1.01-1.49** | **0.038** | 1.05 | 0.86-1.28 | 0.651 | **1.34** | **1.07-1.67** | **0.012** | 1.03 | 0.80-1.33 | 0.800 |
| Weekly | **1.35** | **1.11-1.65** | **0.003** | 1.10 | 0.89-1.36 | 0.375 | 1.19 | 0.93-1.51 | 0.168 | 0.89 | 0.68-1.16 | 0.386 |
| **Hobby/project** | | | | | | | | | | | | |
| Monthly | **0.86** | **0.76-0.97** | **0.014** | 0.99 | 0.86-1.13 | 0.891 | **0.85** | **0.73-0.99** | **0.037** | 1.02 | 0.85-1.22 | 0.829 |
| Weekly | **0.84** | **0.74-0.96** | **0.008** | 0.98 | 0.85-1.14 | 0.827 | 0.87 | 0.74-1.01 | 0.073 | 1.08 | 0.90-1.29 | 0.420 |
| **Club** | | | | | | | | | | | | |
| Monthly | **0.71** | **0.62-0.80** | **<0.001** | **0.83** | **0.72-0.95** | **0.007** | **0.66** | **0.56-0.77** | **<0.001** | **0.79** | **0.66-0.95** | **0.013** |
| Weekly | **0.64** | **0.54-0.75** | **<0.001** | **0.77** | **0.65-0.91** | **0.003** | **0.60** | **0.50-0.72** | **<0.001** | **0.70** | **0.57-0.86** | **0.001** |
| **Organization** | | | | | | | | | | | | |
| Monthly | **0.82** | **0.70-0.96** | **0.011** | 0.87 | 0.74-1.03 | 0.108 | **0.80** | **0.66-0.97** | **0.020** | 0.87 | 0.71-1.07 | 0.196 |
| Weekly | 1.04 | 0.80-1.34 | 0.787 | 1.02 | 0.76-1.36 | 0.902 | 1.18 | 0.90-1.55 | 0.239 | 1.23 | 0.91-1.66 | 0.180 |

*Note.* For all activities, no engagement was the reference category. In both concurrent and longitudinal analyses, model 2 was adjusted for gender, race/ethnicity, education, age, marital status, employment status, household income, neighborhood safety, social network, difficulty with ADLs and IADLs, long-term conditions, and cognition. Longitudinal model 2 was additionally adjusted for depression in the previous wave. Results weighted and based on 20 multiply imputed data sets. Bold text indicates p<0.05.

### Inclusion of 2018 HRS data

In our main analyses, we used data from HRS waves at which the frequency of arts engagement was consistently measured, and complete data were available (2008-2016). An early release file also provides preliminary data from the HRS 2018 wave. This data could not be included in our main analyses because variables indicating eligibility for and completion of the Psychosocial and Lifestyle Questionnaire in 2018 were not available, and neither were weights to account for non-response to the Psychosocial and Lifestyle questionnaire in 2018. However, as our main analyses used HRS weights for the first year in which participants completed the Psychosocial and Lifestyle questionnaire, we were able to include individuals who participated in 2018 and had also participated in the Psychosocial and Lifestyle questionnaire in a previous year. Concurrent models included arts engagement and depression measured simultaneously, with estimates averaged across all waves (2008-2018). In longitudinal models, exposures (arts engagement) were included from 2008 to 2016, with the outcome (depression) included from 2010 to 2018.

Data on difficulties with ADLS and IADLS and cognition were not available in 2018. For these analyses, confounders were thus limited to gender, race/ethnicity, education, age, marital status, employment status, household income, neighborhood safety, social network, and long-term conditions (as well as depression in the previous wave for longitudinal analyses).

Table S9. *Models testing associations between frequency of leisure activities and the odds of depression (indicated by a score of 3 or more on the CES-D) including data from the 2018 HRS wave.*

|  | **Concurrent** (n=19,176) | | | | | | **Longitudinal** (n=15,952) | | | | | |
| --- | --- | --- | --- | --- | --- | --- | --- | --- | --- | --- | --- | --- |
|  | **Model 1: Unadjusted** | | | **Model 2: Adjusted** | | | **Model 1: Unadjusted** | | | **Model 2: Adjusted** | | |
|  | OR | 95% CI | p value | OR | 95% CI | p value | OR | 95% CI | p value | OR | 95% CI | p value |
| **Reading** | | | | | | | | | | | | |
| Monthly | **0.70** | **0.62-0.80** | **<0.001** | **0.85** | **0.74-0.99** | **0.034** | **0.72** | **0.62-0.83** | **<0.001** | 0.94 | 0.78-1.14 | 0.544 |
| Weekly | **0.56** | **0.49-0.62** | **<0.001** | **0.77** | **0.68-0.87** | **<0.001** | **0.54** | **0.47-0.62** | **<0.001** | **0.80** | **0.68-0.95** | **0.009** |
| **Writing** | | | | | | | | | | | | |
| Monthly | 1.05 | 0.97-1.14 | 0.257 | 1.04 | 0.95-1.14 | 0.374 | 0.97 | 0.88-1.06 | 0.506 | 0.91 | 0.81-1.02 | 0.114 |
| Weekly | 1.00 | 0.91-1.10 | 0.968 | 1.03 | 0.93-1.14 | 0.598 | 1.03 | 0.93-1.14 | 0.632 | 1.03 | 0.91-1.17 | 0.627 |
| **Baking/cooking** | | | | | | | | | | | | |
| Monthly | 1.02 | 0.94-1.10 | 0.651 | 0.93 | 0.86-1.01 | 0.105 | 1.04 | 0.95-1.13 | 0.406 | 0.95 | 0.85-1.06 | 0.381 |
| Weekly | **0.92** | **0.86-1.00** | **0.045** | **0.82** | **0.76-0.90** | **<0.001** | 0.96 | 0.88-1.04 | 0.309 | **0.88** | **0.79-0.97** | **0.012** |
| **Sewing** | | | | | | | | | | | | |
| Monthly | 1.06 | 0.93-1.21 | 0.383 | 0.89 | 0.78-1.03 | 0.119 | **1.16** | **1.00-1.34** | **0.046** | 1.00 | 0.83-1.20 | 0.993 |
| Weekly | **1.29** | **1.12-1.48** | **<0.001** | 1.01 | 0.87-1.17 | 0.927 | **1.19** | **1.02-1.39** | **0.028** | 0.86 | 0.72-1.04 | 0.121 |
| **Hobby/project** | | | | | | | | | | | | |
| Monthly | **0.63** | **0.58-0.68** | **<0.001** | **0.76** | **0.70-0.83** | **<0.001** | **0.74** | **0.67-0.80** | **<0.001** | 0.98 | 0.87-1.10 | 0.728 |
| Weekly | **0.62** | **0.57-0.68** | **<0.001** | **0.76** | **0.69-0.83** | **<0.001** | **0.60** | **0.54-0.66** | **<0.001** | **0.80** | **0.71-0.90** | **<0.001** |
| **Club** | | | | | | | | | | | | |
| Monthly | **0.69** | **0.64-0.75** | **<0.001** | **0.81** | **0.74-0.89** | **<0.001** | **0.68** | **0.62-0.74** | **<0.001** | **0.79** | **0.71-0.89** | **<0.001** |
| Weekly | **0.61** | **0.55-0.68** | **<0.001** | **0.72** | **0.65-0.81** | **<0.001** | **0.63** | **0.56-0.70** | **<0.001** | **0.80** | **0.70-0.92** | **0.001** |
| **Organization** | | | | | | | | | | | | |
| Monthly | **0.89** | **0.81-0.99** | **0.029** | 0.95 | 0.85-1.06 | 0.351 | **0.83** | **0.74-0.92** | **0.001** | **0.85** | **0.74-0.98** | **0.021** |
| Weekly | 1.14 | 0.97-1.35 | 0.111 | 1.13 | 0.94-1.35 | 0.181 | 1.09 | 0.91-1.29 | 0.354 | 1.03 | 0.83-1.29 | 0.765 |

*Note.* For all activities, no engagement was the reference category. In both concurrent and longitudinal analyses, model 2 was adjusted for gender, race/ethnicity, education, age, marital status, employment status, household income, neighborhood safety, social network, and long-term conditions. Longitudinal model 2 was additionally adjusted for depression in the previous wave. Results weighted and based on 20 multiply imputed data sets. Bold text indicates p<0.05.
